# Comparative Study on Supercapacitive Performances of Hierarchically Nanoporous Carbon Materials With Morphologies From Submicrosphere to Hexagonal Microprism

**DOI:** 10.3389/fchem.2020.599981

**Published:** 2020-11-17

**Authors:** Lei Xie, Kai Yuan, Jianxiong Xu, Yirong Zhu, Lijian Xu, Na Li, Jingjing Du

**Affiliations:** ^1^College of Packaging and Material Engineering, Hunan University of Technology, Zhuzhou, China; ^2^Hunan Key Laboratory of Biomedical Nanomaterials and Devices, College of Life Sciences and Chemistry, Hunan University of Technology, Zhuzhou, China; ^3^Hunan Key Laboratory of Electrochemical Green Metallurgy Technology, College of Metallurgy and Materials Engineering, Hunan University of Technology, Zhuzhou, China; ^4^National and Local Joint Engineering Research Center of Advanced Packaging Materials Developing Technology, Hunan University of Technology, Zhuzhou, China

**Keywords:** supercapacitor, hierarchically nanoporous carbon, electrode materials, well-defined morphologies, controllable mesostructures

## Abstract

Hierarchically nanoporous carbon materials (HNCMs) with well-defined morphology and excellent electrochemical properties are promising in fabrication of energy storage devices. In this work, we made a comparative study on the supercapacitive performances of HNCMs with different morphologies. To this end, four types of HNCMs with well-defined morphologies including submicrospheres (HNCMs-S), hexagonal nanoplates (HNCMs-N), dumbbell-like particles (HNCMs-D), and hexagonal microprisms (HNCMs-P) were successfully synthesized by dual-template strategy. The relationship of structural–electrochemical property was revealed by comparing the electrochemical performances of these HNCMs-based electrodes using a three-electrode system. The results demonstrated that the HNCMs-S–based electrode exhibited the highest specific capacitance of 233.8 F g^−1^ at the current density of 1 A g^−1^ due to the large surface area and well-defined hierarchically nanoporous structure. Moreover, the as-prepared HNCMs were further fabricated into symmetrical supercapacitor devices (HNCMs-X//HNCMs-X) using KOH as the electrolyte and their supercapacitive performances were checked. Notably, the assembled HNCMs-S//HNCMs-S symmetric supercapacitors displayed superior supercapacitive performances including high specific capacitance of 55.5 F g^−1^ at 0.5 A g^−1^, good rate capability (retained 71.9% even at 20 A g^−1^), high energy density of 7.7 Wh kg^−1^ at a power density of 250 W kg^−1^, and excellent cycle stability after 10,000 cycles at 1 A g^−1^. These results further revealed the promising prospects of the prepared HNCMs-S for high-performance energy storage devices.

## Introduction

With the development of industry and economy of human society, environmental and energy problems have been becoming more and more serious. Developing a new energy of environmental protection and pollution-free and achieving greater energy efficiency are the important means to solve this problem (Liu Z. X. et al., 2016; Qi et al., 2019; Yu Z. L. et al., 2019; Zhu et al., 2019a,b). Supercapacitor as an environmentally friendly energy storage device has been demonstrated to be superior to traditional capacitors and rechargeable batteries, due to the high power density, fast storage and release energy rate, and long cycle stability (Xie et al., 2016, 2018; Hossain et al., 2017; Zhang Z. T. et al., 2017; Yao et al., 2018; Wei et al., 2020a). In principle, supercapacitors can be divided into electronic double-layer capacitors (EDLCs) and pseudocapacitors. Compared with pseudocapacitors, the energy storage mechanism of EDLCs is via physical accumulation of charge at the electrode–electrolyte interface, which will result in fast charge–discharge rates and long lifetimes (Wang L. L. et al., 2017; Guo et al., 2018; Chen et al., 2019; Muzaffara et al., 2019; Lv et al., 2020). During the past few decades, bulk carbon materials including active carbon (Lee et al., 2017; Zhang X. R. et al., 2019), mesoporous carbon (Bo et al., 2019; Wei et al., 2020b,c), graphene (El-Kady et al., 2016; Wang F. X. et al., 2017), carbon nanotube (Afzal et al., 2017; Zhang et al., 2017), and carbide-derived carbon (Dyatkin et al., 2016; Brousse et al., 2017) have been extensively documented as electrode materials for EDLCs. Although these carbon-based EDLCs exhibit high power density and rapid charge–discharge rate, the unsatisfactory energy density of carbon-based SCs (4–5 Wh kg^−1^) was still a limitation (Chang et al., 2017; Yang and Zhou, 2017; Wan et al., 2019).

Recently, porous carbon materials have been recognized as more promising electrode materials of supercapacitors in view of their excellent electric conductivity, high surface area, and stable chemical and thermal stability (Li et al., 2018; Pang et al., 2018; Yu J. G. et al., 2019; Yu et al., 2020). According to the pore size, porous carbon materials can be classified into microporous, mesoporous, and macroporous carbon materials. It has been found that different dimensions of pore have unique contribution to the electrochemical performance of the porous carbon material. Generally, the micropores enhance the electrical double-layer capacitance, the mesopores provide ion transport pathways with minimized resistance, and the macropores serve as ion-buffering reservoirs to reduce the diffusion distance (Xiong et al., 2017; Guan et al., 2018; Wu et al., 2018). Nevertheless, carbon materials with single pore structure have its own restriction in fabrication of high performance of supercapacitors. On the contrary, hierarchically porous carbon materials possess several advantages: (1) more accessible surface area by virtue of the coexistence of micropores and mesopores/macropores, which leads to the enhanced surface area and improvement specific capacitance; and (2) the unique hierarchically porous architecture provides continuous electrolyte ion diffusion pathways, resulting in electroactive sites effective and enhanced rate performance (Liu et al., 2017, 2020; Li J. Y. et al., 2019). Until now, bimodal or trimodal porous carbon materials, including micro–meso (Yang et al., 2018; Zhu B. X. et al., 2019), meso–meso (Wang L. et al., 2019), meso–macro (Yu Q. et al., 2019), and even micro–meso–macro (Wang Q. et al., 2014; Zhang et al., 2017) materials have been synthesized and applied in supercapacitors. Comparatively speaking, hierarchically nanoporous carbon materials (HNCMs) with interconnected micro–meso–macro pore architecture are more promising because of the synergistic effect of pores in different levels (Luo et al., 2018; Sheng et al., 2018a,b; Tan et al., 2019).

Besides the pore structure, the morphological features of the porous carbon materials are another important factor that affects the electrochemical performance because porous carbon materials with unique morphology can significantly increase the contact area between the electrode materials and electrolyte (Guo et al., 2015; Shieh et al., 2017; Feng et al., 2019; Shi et al., 2020). Several meaningful research works have put eyes on tailoring the morphology of porous carbon materials and their related electrochemical performances. For instance, Xie et al. (2019) reported the controlled synthesis of ordered mesoporous carbon nanoparticles with morphology involved from rhombic dodecahedron to spheres by adjusting the amount of soft template F127. Li and Xue (2012) used a facile soft template to synthesize highly ordered mesoporous carbon nanoparticles with controllable morphology from spherical to worm-like and rod-shaped. Electrochemical measurements showed that the spherical mesoporous carbon with diameter of 200 nm exhibited excellent capacitance of about 142 F g^−1^ at current density of 0.5 A g^−1^ due to its highly ordered mesochannels, larger specific surface area, and pore volume. Qiu et al. (2018) synthesized hierarchical porous hollow carbon with morphologies involving from hollow sphere to bowl by adjusting the KOH dosage. The porous hollow carbon spheres and carbon bowls were utilized as the anode and cathode, respectively, in fabrication of sodium-ion hybrid capacitors. As far as we are concerned, studies on the morphological effect of HNCMs on its electrochemical performance have been seldom reported.

In this contribution, four types of HNCMs with different morphologies of submicrospheres, hexagonal nanoplates, dumbbell-like particles, and hexagonal microprisms were controlled synthesized via dual-template strategy. The morphological and structural properties of these four samples were comparatively studied. Moreover, the supercapacitive performances of HNCMs with different morphologies were compared.

## Results and Discussion

### Morphological and Structural Characterization of HNCMs

HNCMs were prepared by dual-template strategy employing hexadecylpyridinium chloride/poly(acrylic acid) (CPC/PAA) organic mesomorphous complexes as dynamic soft template, the *in situ*–generated silica as hard template, tetraethylsiloxane (TEOS) as silica source, and sucrose as carbon precursor as reported in our previous work (Xu et al., 2020). By varying the adding amount of PAA in the synthesis, a series of HNCMs were synthesized. The samples were denoted as HNCMs-S, HNCMs-N, HNCMs-D, and HNCMs-P, depending on the added amount of PAA at 4.0, 4.9, 5.3, and 5.8 g, respectively.

**Figure 1:**
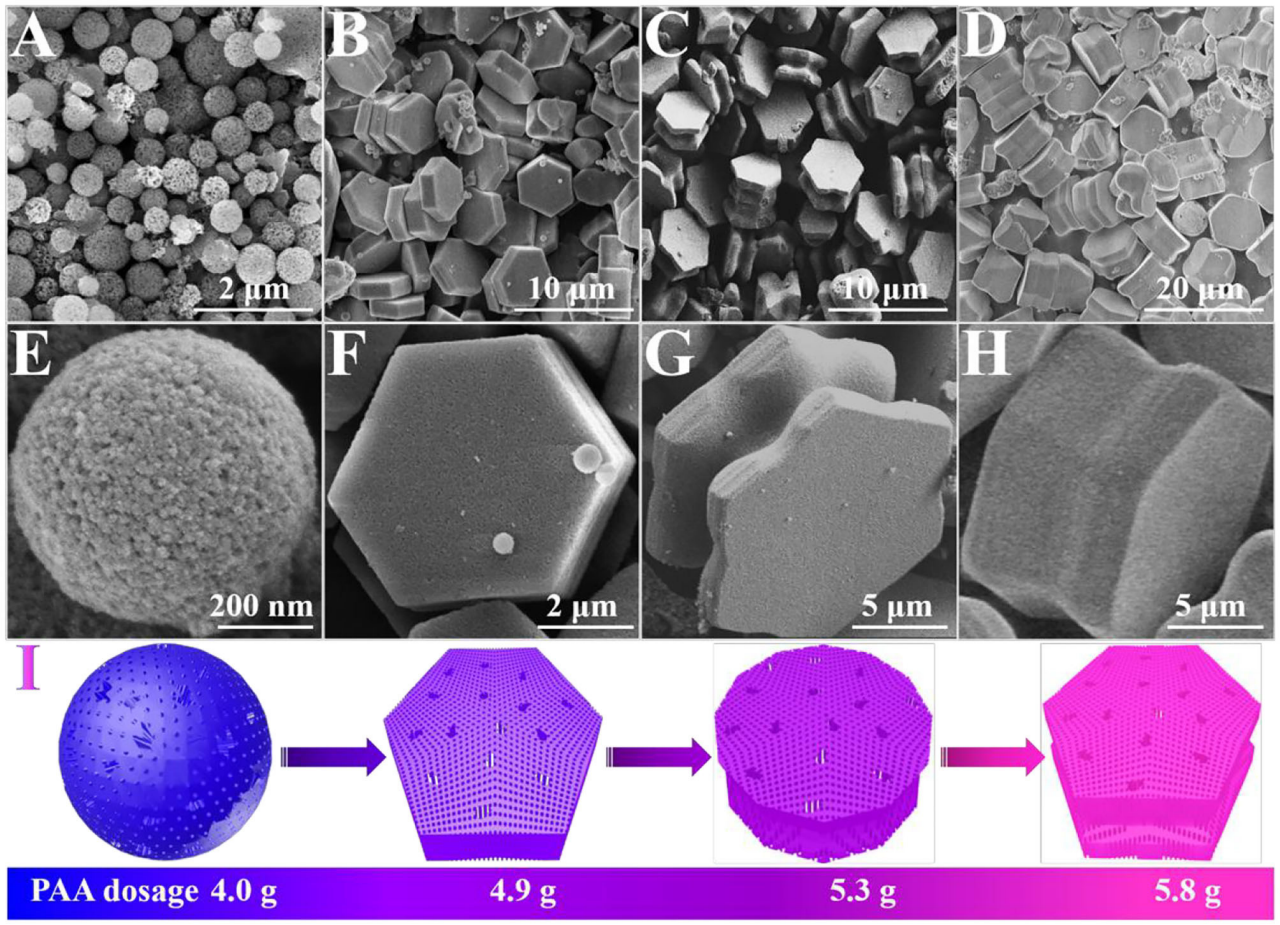
FE-SEM images of the HNCM samples synthesized with different amount of PAA: **(A,E)** HNCMs-S, **(B,F)** HNCMs-N, **(C,G)** HNCMs-D, **(D,H)** HNCMs-P, and **(I)** models of morphologies change in these samples.

The morphological difference of these samples was revealed by field emission scanning electron microscopy (FE-SEM). As shown in Figure 1A, the sample HNCMs-S presented as uniform submicrometer spherical particles with the average diameter of ~600 nm. The high-magnification FE-SEM image of Figure 1E reveals that the HNCMs-S possesses a rough surface and obviously hierarchical porous architecture. The interior texture of the HNCMs-S particle was characterized by transmission electron microscopy (TEM), and the results are shown in Supplementary Figure 1. It can be seen that HNCMs-S particle contains interstitial nanopores, as well as ordered mesopores. When the adding amount of PAA was increased to 4.9 g, uniform well-defined hexagonal carbon nanoplates (HNCMs-N) could be obtained. These nanoplates are about 4 μm in diameter and 500 nm in average thickness (Figures 1B,F). Further increasing the PAA amount to 5.3 g, it is interesting that the obtained HNCMs-D exhibited a dumbbell-like morphology with two hexagonal nanoplates at each side and a sunken area at the center. The size of the hexagonal nanoplate at each side is similar and measured to be about 5 μm, as shown in Figures 1C,G. Uniform hexagonal carbon microprism (HNCMs-P) with a diameter of ~7 μm and height of ~5 μm (Figures 1D,H) was obtained when the PAA amount was fixed at 5.8 g. These results confirm that the added amount of PAA in the synthesis has a significant impact on the morphology of the synthesized HNCMs. By varying the adding amount of PAA in the synthesis, synthesis of hierarchically nanoporous carbon with novel morphologies such as microspheres, hexagonal nanoplate, dumbbell-like, and hexagonal microprism can be controlled, as schematically shown in Figure 1I. The TEM images of the HNCMs-N, HNCMs-D, and HNCMs-P samples were not given because the particle sizes were too large to be observed by TEM.

**Figure 2:**
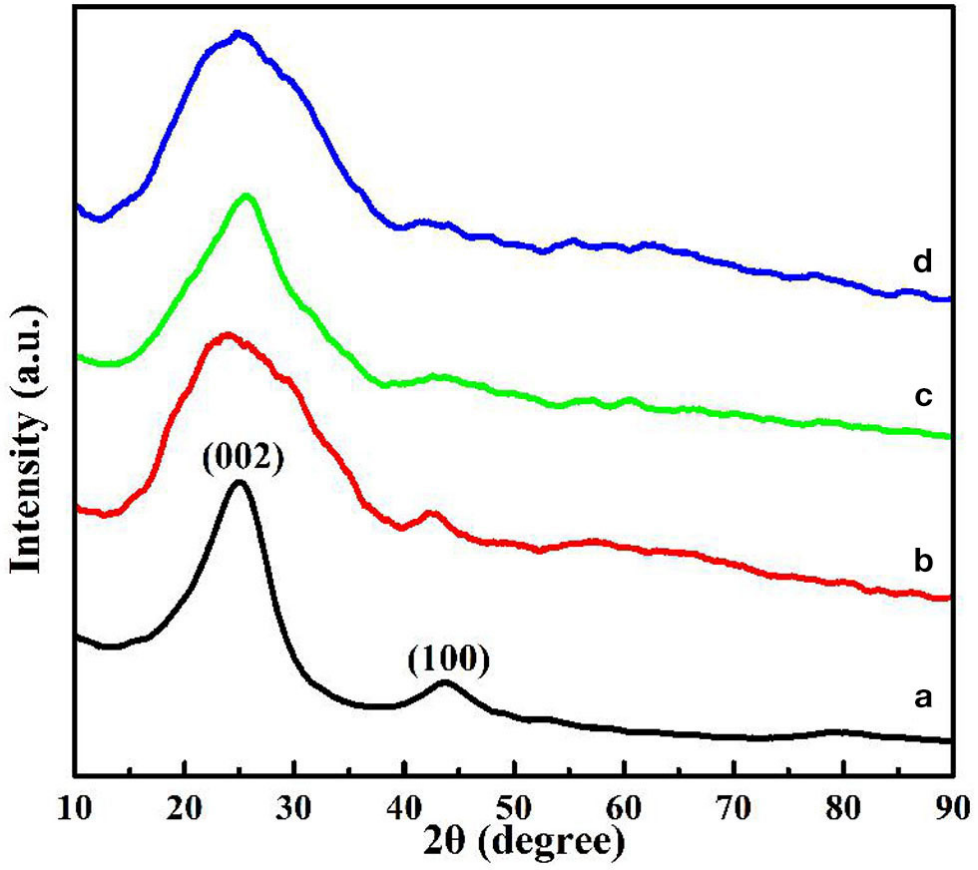
X-ray diffraction patterns of the HNCMs synthesized with different amount of PAA: HNCMs-S (pattern a), HNCMs-N (pattern b), HNCMs-D (pattern c), and HNCMs-P (pattern d).

The crystalline structure of the as-obtained HNCMs materials was further compared by powder X-ray diffraction (XRD) measurement. As shown in Figure 2, the XRD patterns of the HNCMs-S displayed a broad diffraction peaks at 2 θ values of 25°, which could be attributed to the hexagonal graphite (002) planes of amorphous carbon (Chang et al., 2016). The broad peak is an indicative of low graphitization degree. Moreover, the intensity of (002) diffraction peak for HNCMs decreased with increasing the PAA amount, indicating the reduction of graphitization degree. Meanwhile, another broad peak at 44° corresponding to the (100) planes, relating to the interlayer condensation of graphite layers at high carbonization temperature (Liu et al., 2018), was also seen in the XRD patterns of HNCMs-S (pattern a) and HNCMs-N (pattern b). Generally, the intensity of (100) diffraction peaks for HNCMs also decreased with increasing the PAA amount. Typically, the diffraction peak at 2 θ values of 44° became unapparent for the XRD patterns of HNCMs-D (pattern c) and HNCMs-P (pattern d). These results indicated that the HNCMs-S possesses a relatively higher graphitization degree, potentially suggesting the better electrical conductivity.

**Figure 3:**
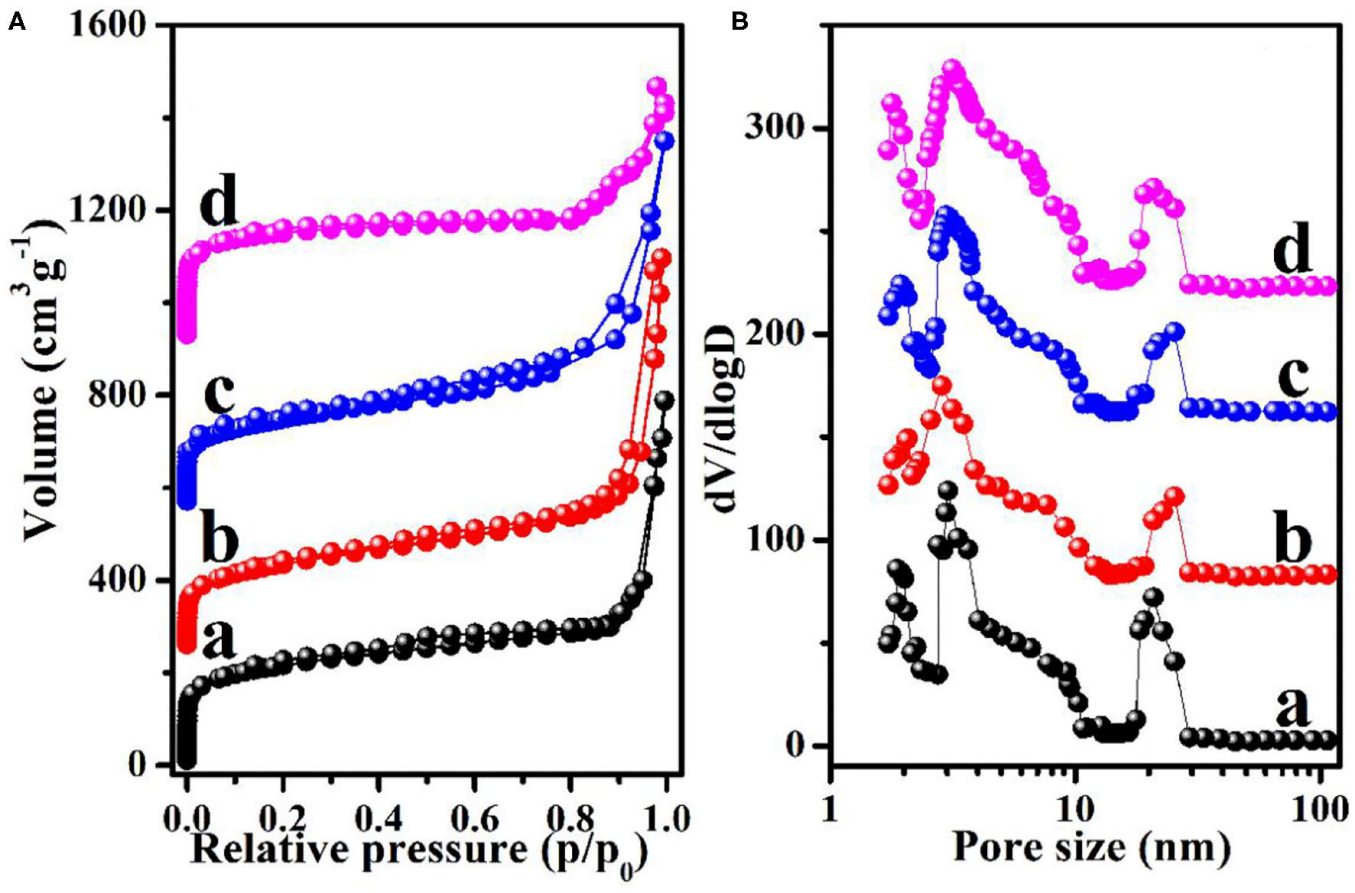
**(A)** The nitrogen adsorption-desorption isotherms and **(B)** DFT pore size distribution curves of the HNCMs synthesized under different amount of PAA: (a) HNCMs-S, (b) HNCMs-N, (c) HNCMs-D, and (d) HNCMs-P.

To further assess the structural differences of the HNCMs materials, the four samples were subjected to N_2_ adsorption–desorption measurement. The N_2_ adsorption–desorption isotherms and pore size distributions curves of the four HNCMs are shown in Figure 3. As shown in Figure 3A, combined I and IV features containing three adsorption steps could be observed from the N_2_ adsorption–desorption curves of all the HNCM samples (Wang S. L. et al., 2017; Wang H. R. et al., 2019). The sharp rise at low relative pressure range (*P*/*P*_0_ < 0.1), revealing the existence of a certain amount of micropores in the HNCM samples. The gradual increased step in the relative pressure region of 0.3–0.8 is the typical characteristic of mesopores. The pronounced hysteresis loop at the relative pressure of 0.9 < *P*/*P*_0_ < 0.99 manifests the existence of large interstitial nanopores. The pore size distribution curves of HNCMs calculated from adsorption branch by using the DFT model is shown in Figure 3B. Three peaks centered at 1.7–1.9, 3.0, and 20–40 nm are observed in each curve of Figure 3B, which further confirmed the hierarchically nanoporous structure with micropores, mesopores, and nanopores of the HNCM samples. The specific surface area and total pore volume of the HNCM samples are tabulated in Table 1. It was clear that HNCMs-S showed the highest specific surface area of 737 m^2^ g^−1^ and the largest pore volume of 1.23 cm^3^ g^−1^, which might be ascribed to the relatively small size and well-developed mesopores and secondary interstitial nanopores.

**Table 1:** Structural parameters of HNCM samples.

**Sample**	**Pore structure parameters**					
	***S*_**BET**_ (m^**2**^ g^**−1**^)**	***S*_**micro**_ (m^**2**^ g^**−1**^)**	***S*_**meso**_ (m^**2**^ g^**−1**^)**	***V*_**total**_ (cm^**3**^ g^**−1**^)**	***V*_**micro**_ (cm^**3**^ g^**−1**^)**	***V*_**meso**_ (cm^**3**^ g^**−1**^)**
HNCMs-S	737	175	562	1.23	0.18	1.05
HNCMs-N	713	159	554	1.05	0.15	0.90
HNCMs-D	674	117	557	0.99	0.08	0.91
HNCMs-P	724	201	523	1.09	0.20	0.89

### Electrochemical Performance of HNCMs

**Figure 4:**
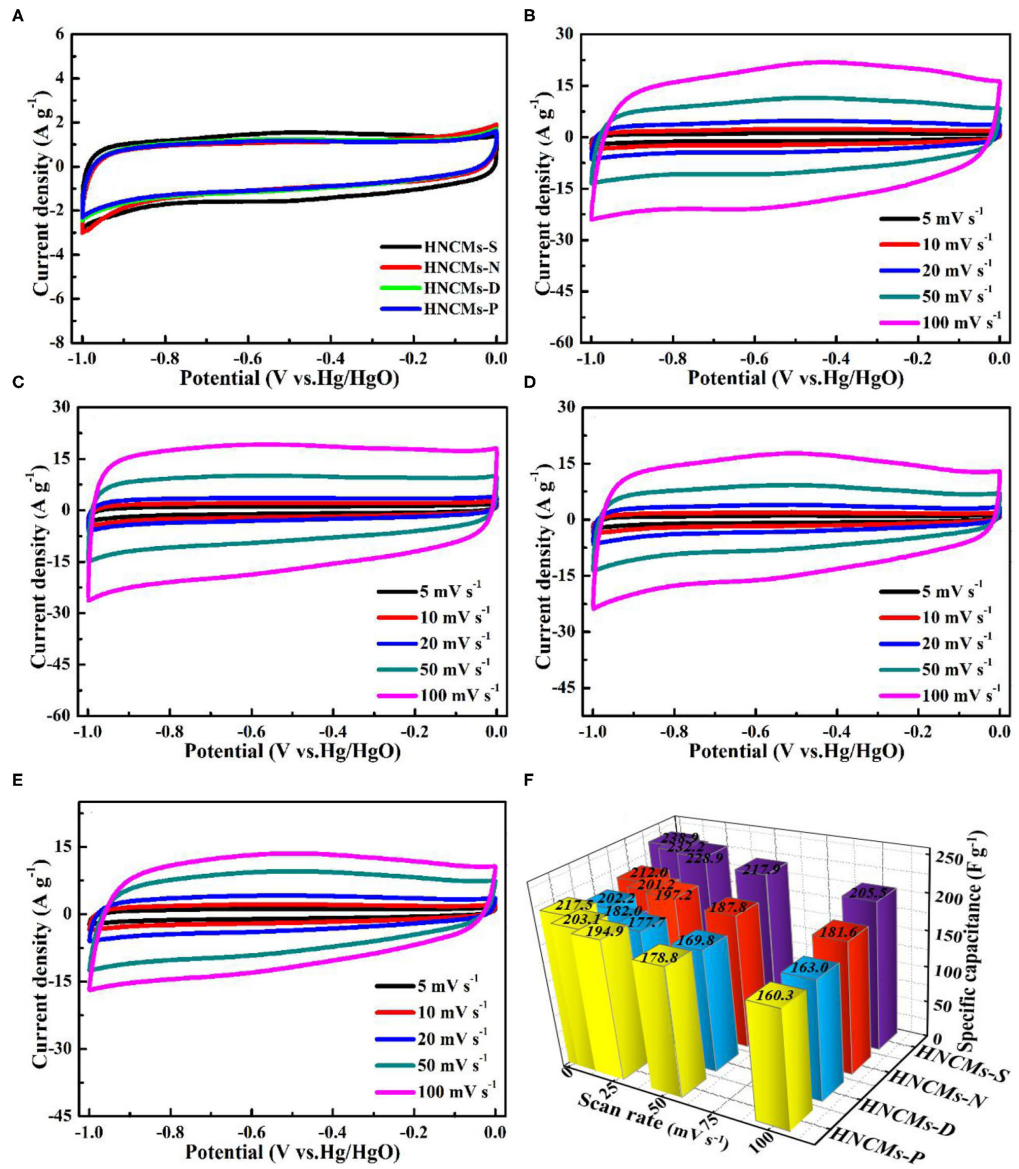
**(A)** CV curves of HNCM electrodes at a scan rate of 5 mV s^−1^, CV curves of HNCM electrodes at different scan rates: **(B)** HNCMs-S, **(C)** HNCMs-N, **(D)** HNCMs-D, and **(E)** HNCMs-P. **(F)** Specific capacitance of the HNCM electrodes as working electrodes at different scan rates.

To compare the electrochemical properties of the as-obtained HNCMs, the four HNCMs were fabricated into electrodes, and the electrochemical performances of the HNCMs-based electrodes were evaluated by cyclic voltammetry (CV), galvanostatic charge–discharge (GCD), and electrochemical impedance spectroscopy measurements using a three-electrode system in 6 M KOH aqueous electrolyte at room temperature. Figure 4A compares the CV curves of the four HNCMs-based electrodes over a potential range from −1.0 to 0 V (vs. Hg/HgO) at a 5 mV s^−1^ scanning rate. As shown, the CV curves of the four HNCMs-based electrodes display similar, well-symmetric quasi-rectangular shape indicating the double-layer capacitive behavior of these HNCMs (Zhu et al., 2018). Moreover, the CV curves of the HNCMs-S electrode encircle a much larger curve area than the other three HNCM electrodes, which is an indicator of high specific capacitance. To make a further comparison, the CV measurements of all the HNCM electrodes at the scanning rate from 5 to 100 mV s^−1^ were further evaluated, and the results are shown in Figures 4B–E. At low scan rate, all the samples exhibit superior rectangular shapes because the electrolyte ions have enough time to move into the porous structure of the HNCMs for the formation of the electrical double layer. However, when the scan rate was increased to 100 mV s^−1^, the CV curves of all the HNCM electrode have a relative obvious peak in the potential range of −0.8 to −0.2 V, which can be attributed to faradaic pseudo-capacitance contributed by redox reactions. This phenomenon may be due to the oxygen-containing functional group [C=O (carbonyl group or quinone group, and carbonxylic group), 1,105 cm^−1^] on the surface of the electrode material as discussed in Supplementary Figure 2, which also have been found in other works (Liu et al., 2016; Liang et al., 2018; Li X. Y. et al., 2019). The specific capacitance of the HNCM electrode–based CV curves at different scanning rate can be calculated by the following equation formula (Zhao et al., 2014):

(1) $$\begin{array}{r} {\text{C}}_{\text{s}}=\left({\text{I}}_{\text{a}}+{\text{I}}_{\text{c}}\right)/2\text{m}\left(d\text{V}/d\text{t}\right) \end{array}$$

where *C*_s_ stands for specific capacitance (F g^−1^), *I*_a_ and *I*_c_ represent the current (A) of anodic and cathodic CV curves on positive and negative sweeps, *m* is the mass of active material (g), and the d*V/*d*t* is the scan rate (mV s^−1^). The results are summarized in Figure 4F. As shown, the HNCMs-S electrode has a higher specific capacitance than the other three electrodes at all scan rates. Under the scan rate of 5 mV s^−1^, the HNCMs-S electrode exhibited the highest capacitance of 238.9 F g^−1^. This might be due to the relative high specific surface area of HNCMs-S, which could facilitate the adsorption of electrolyte ions.

**Figure 5:**
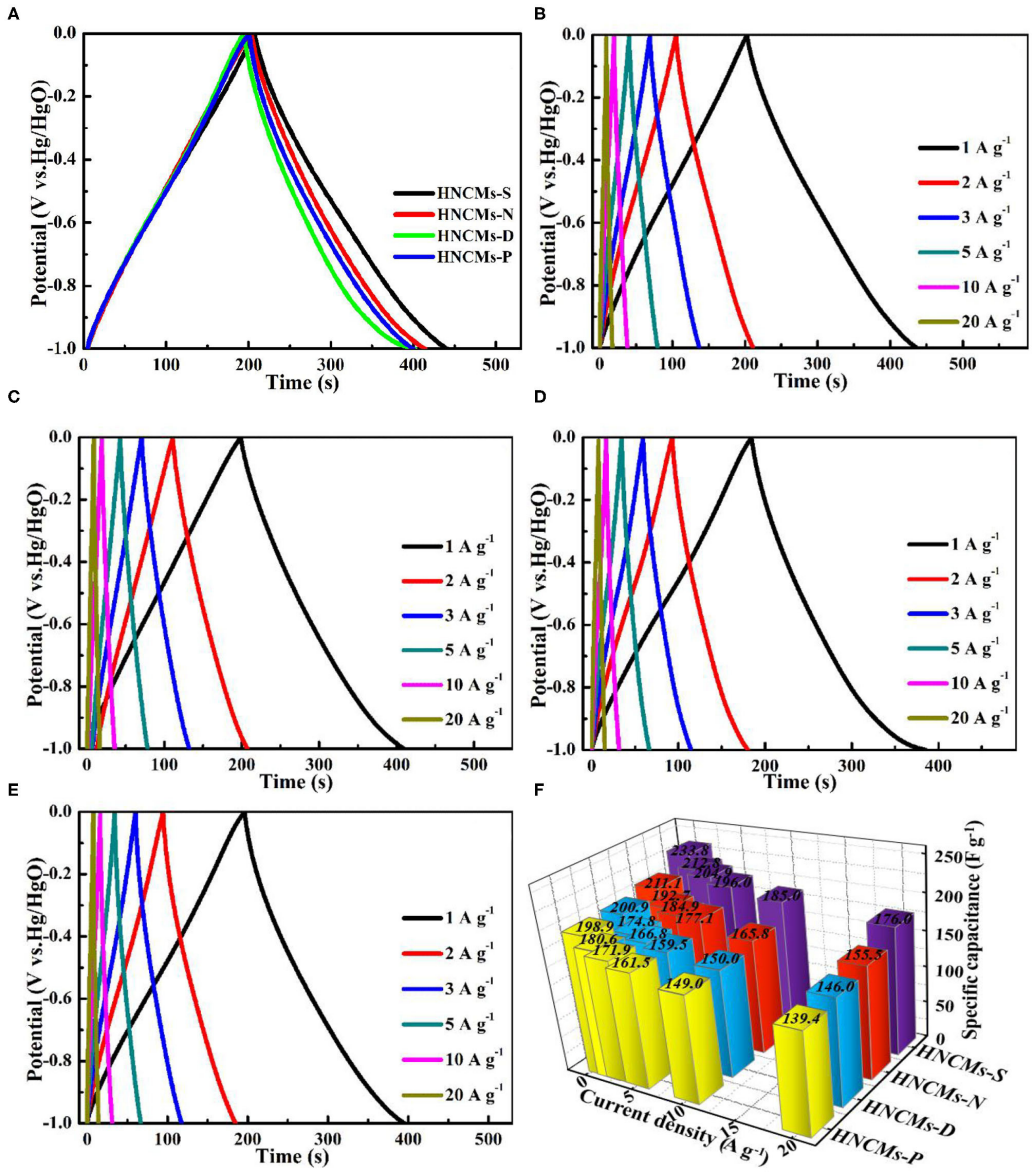
**(A)** Constant current charge and discharge curves of different HNCM electrodes at current density of 1 A g^−1^, constant current charge and discharge curves of HNCM samples at different current densities: **(B)** HNCMs-S, **(C)** HNCMs-N, **(D)** HNCMs-D and **(E)** HNCMs-P. **(F)** Specific capacitance of the HNCM electrodes as working electrodes at different current densities.

The GCD profiles of the HNCM electrodes between −1 and 0 V were also evaluated in order to further confirm the reversible reaction of the HNCM electrodes, as illustrated in Figure 5. To make comparison, we initially compared the GCD curves of the HNCM electrodes at the current density of 1 A g^−1^. As shown in Figure 5A, all the GCD curves of the four electrodes present approximately isosceles triangular shape, implying the ideal capacitive performance and outstanding electrochemical reversibility of the HNCM electrode material (Figure 5A) (Cao et al., 2019). Obviously, the HNCMs-S electrode has longer discharging time than other HNCM electrodes, revealing its relatively higher specific capacitance. The specific capacitance of the HNCM electrode–based GCD curve can be calculated according to the following equation:

(2) $$\begin{array}{r} {\text{C}}_{\text{m}}={\text{it}}_{\text{d}}/\text{m}\Delta \text{V} \end{array}$$

where *C*_m_ is specific capacitance (F g^−1^), *i* stands for the constant discharging current (A), *t*_*d*_ is the discharging time (s), and *m* represent the mass of the electroactive material in the electrode (g), Δ*V* stands for the potential range of the charge–discharge (V) (Zhao et al., 2015). The specific capacitance of HNCMs-S electrode is calculated to be 233.8 F g^−1^ at the current density of 1 A g^−1^, which is higher than that of the HNCMs-N (211.1 F g^−1^), HNCMs-D (200.9 F g^−1^), and HNCMs-P (198.9 F g^−1^) electrodes (Figure 5F). Moreover, the GCD curves of HNCM electrodes at current density from 1 to 20 A g^−1^ are also presented in Figures 5B–E. All curves exhibit the symmetrical triangular shapes, reconfirming the splendid coulombic efficiency even in the rapid charge–discharge process. In addition, no obvious IR drop in Figures 5B–E can be observed even at high current density of 20 A g^−1^, indicating the little equivalent series resistance of HNCMs. The specific capacitances calculated by GCD curves are summarized in Figure 5F. In all cases, the specific capacitance of HNCMs-S electrode is higher than that of the other HNCM electrode. The result is consistent with CV measurement. In addition, the specific capacitance retention rates for HNCMs-S, HNCMs-N, HNCMs-D, and HNCMs-P electrodes are 75.3, 73.7, 72.7, and 70.1%, respectively, showing the good rate capability of all the HNCM electrodes.

**Figure 6:**
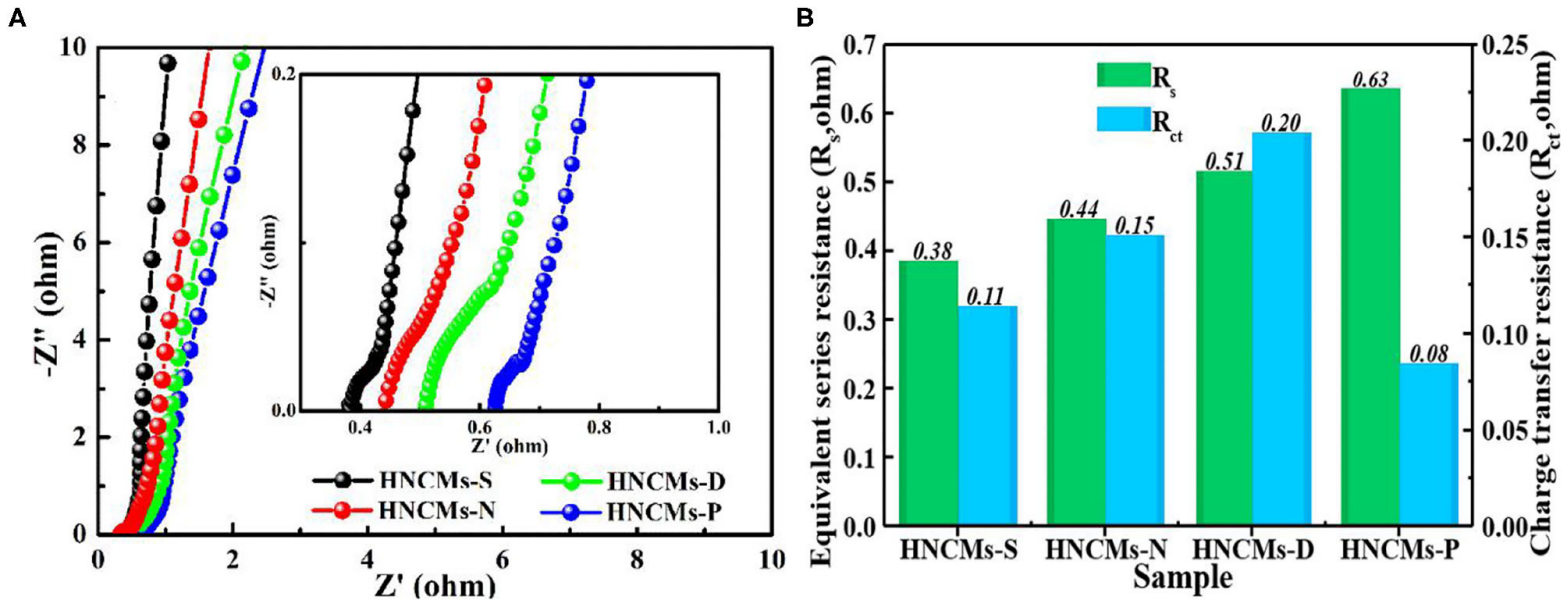
**(A)** Nyquist plots of HNCM electrodes and the inset image shows the magnified curve in the high-frequency region. **(B)** *R*_s_ and *R*_ct_ values of the HNCM electrodes.

We further compared the electrochemical impedance of the HNCM electrodes by electrochemical impedance spectroscopy. Figure 6A displays the Nyquist plots of all HNCM electrodes in 6 M KOH aqueous solution with a frequency range from 10^5^ Hz to 10^−2^ Hz. As shown, all the Nyquist plots of all the four HNCM electrodes show a similar shape, which is composed of a depressed semicircle and a vertical line, as indicated in the inset of Figure 6A. According to the first intersection point of the semicircle with the real *Z* axis (*X* axis) and the diameter of the quasi-semicircle loop at high-frequency region, the equivalent series resistances (*R*_s_) and charge transfer resistance (*R*_ct_) of the four electrodes are measured. The results are displayed in columnar diagram in Figure 6B. As shown, the HNCMs-S electrode exhibited the relative smaller *R*_s_ of 0.38 Ω and *R*_ct_ of 0.11 Ω, indicating rapid electron transfer and superior charge-transfer kinetics in HNCMs-S. Besides, judged from the straight lines in the low-frequency region, HNCMs-S electrode has the smallest migration resistance of the KOH electrolyte ions in the micropores/mesopores.

### Fabrication of HNCMs-Based Supercapacitors and Their Supercapacitive Performances

The HNCM electrodes were further fabricated into symmetrical supercapacitor by using 6 M KOH as the electrolyte and polypropylene as separated membrane. The four symmetrical supercapacitors were named as HNCMs-S//HNCMs-S, HNCMs-N//HNCMs-N, HNCMs-D//HNCMs-D, and HNCMs-P//HNCMs-P, respectively. As shown in Supplementary Figure 3, the CV curves of all the HNCMs-X//HNCMs-X supercapacitors maintained quasi-rectangular shape from 5 mV s^−1^ to high scan rate of 100 mV s^−1^ (Supplementary Figures 3A–E), demonstrating an ideal EDLC behavior. The higher specific capacitance of HNCMs-S//HNCMs-S supercapacitor (74.2 F g^−1^ at 5 mV s^−1^) than the other three HNCMs-X//HNCMs-X supercapacitors can be attributed to the larger surface area and smaller particle size (Supplementary Figure 3F). The GCD curves of HNCMs-based supercapacitor even at high current density of 20 A g^−1^ (Supplementary Figures 4A–E) still exhibit the symmetrical triangular shapes, implying an outstanding electrochemical reversibility and splendid coulombic efficiency. The HNCMs-S//HNCMs-S supercapacitor showed the highest specific capacitance (55.5 F g^−1^ at 0.5 A g^−1^) and rate performance of 71.9% due to its superior hierarchical porous structure and high surface area. The Nyquist plots in Supplementary Figure 5 show that the HNCMs-S//HNCMs-S supercapacitor possessed relative lower internal resistance (*R*_s_) of 0.79 Ω and smaller charge transfer resistance (*R*_ct_) of 0.30 Ω than other HNCMs-X//HNCMs-X supercapacitors, which was mainly due to the good conductivity of the HNCMs-S and high ion-diffusion efficiency of the hierarchical porous architecture.

In view of application, energy density and power density are two important evaluating indicators. According to the GCD profiles, the energy density could be estimated from the following equation:

(3) $$\begin{array}{r} \text{E}={\text{CV}}^{2}/2\times 3.6 \end{array}$$

where *E* is the energy density (Wh kg^−1^), *C* is the specific capacitance of supercapacitor based on CV curve (F g^−1^), and *V* represents the potential window (V) (Zhang Y. X. et al., 2019).

**Figure 7:**
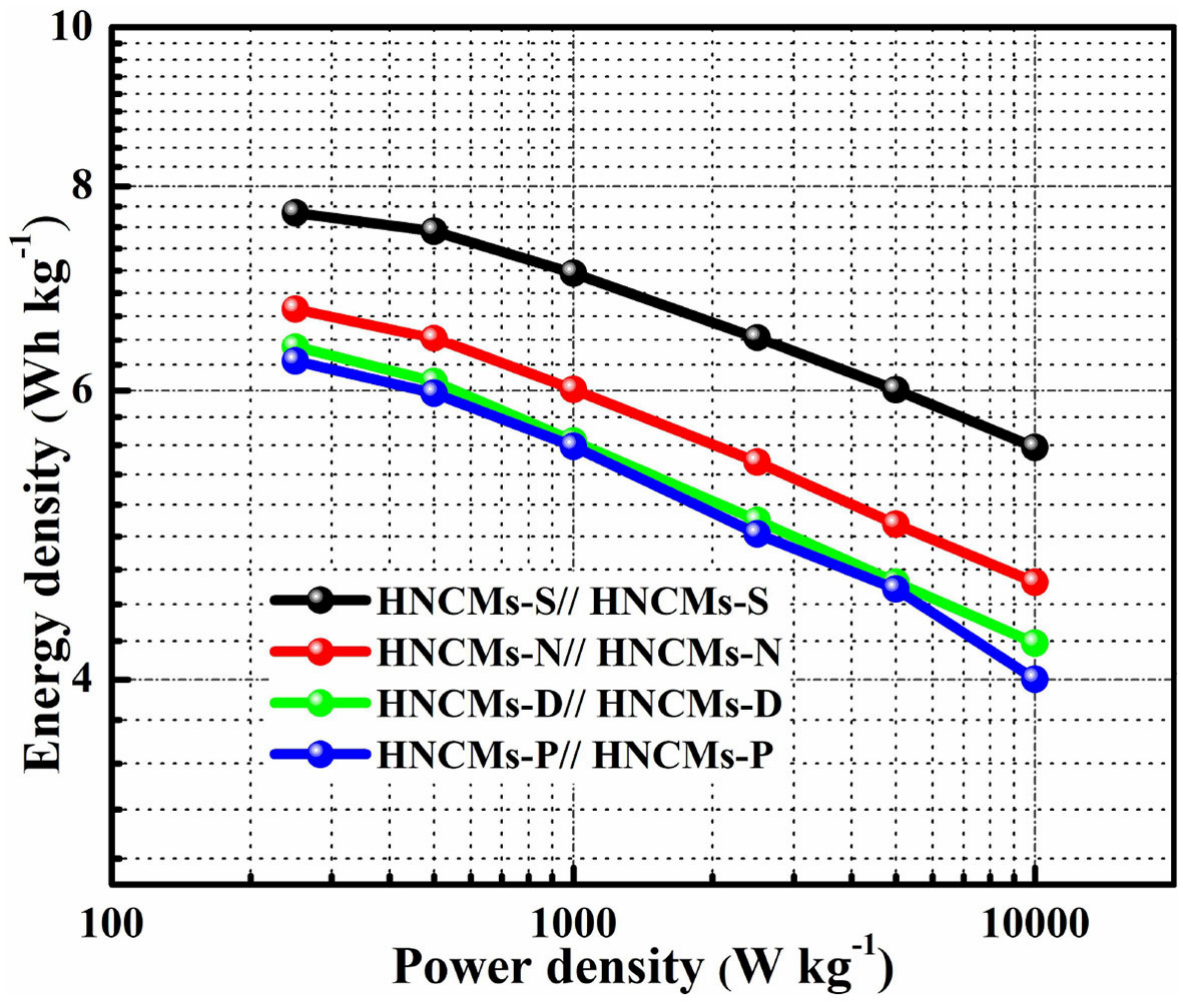
Ragone plots of HNCMs-X//HNCMs-X supercapacitors.

Power density could be estimated using the following equation:

(4) $$\begin{array}{r} \text{P}=3,600\;\times \;\text{E}/\Delta \text{t} \end{array}$$

where *P* is the power density (kW kg^−1^), and Δ*t* represents the discharge time (s) (Ramachandran et al., 2018). The Ragone plots of HNCMs-X//HNCMs-X supercapacitors are shown in Figure 7. It was obvious that the HNCMs-S//HNCMs-S supercapacitor presented the highest energy density at the same power density, which might be ascribed to the well-developed mesopores and high specific surface area. It can be seen that the HNCMs-S//HNCMs-S supercapacitors deliver a high energy density of 7.7 Wh kg^−1^ at power density of 250 W kg^−1^, which is higher than that of the HNCMs-N//HNCMs-N (6.7 Wh kg^−1^), HNCMs-D//HNCMs-D (6.4 Wh kg^−1^), and HNCMs-P//HNCMs-P (6.3 Wh kg^−1^) supercapacitors. Obviously, the energy density of HNCMs-X//HNCMs-X supercapacitors is much higher than that of the commercial activated carbon–based symmetric supercapacitors (3–5 Wh kg^−1^). Notably, an energy density of 5.5 Wh kg^−1^ of the HNCMs-S//HNCMs-S supercapacitor can be retained at a high power density of 10,000 W kg^−1^.

**Figure 8:**
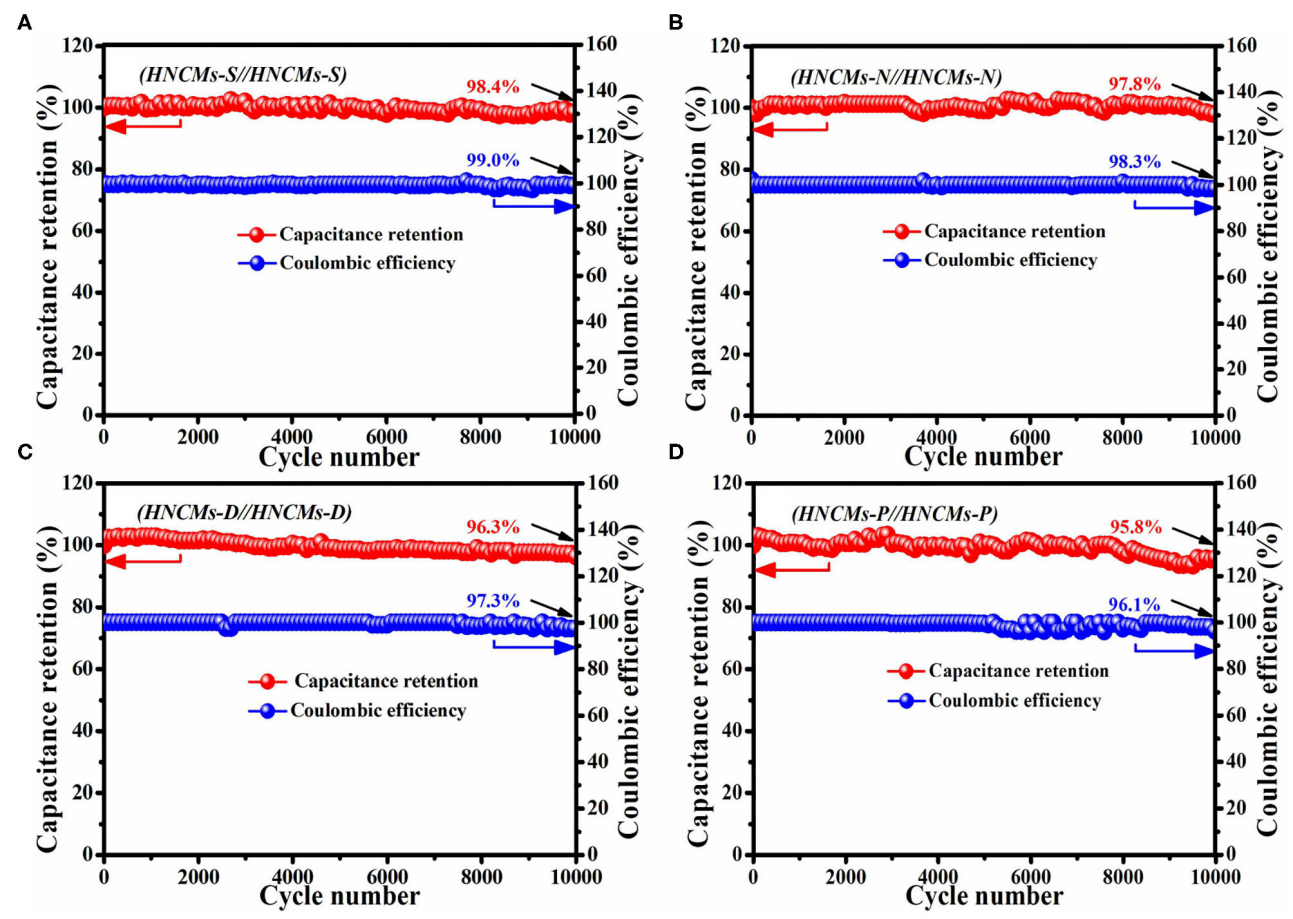
The capacitance retention and coulombic efficiency of HNCMs-X//HNCMs-X supercapacitors at 1 A g^−1^ over 10,000 cycles. **(A)** HNCMs-S//HNCMs-S, **(B)** HNCMs-N//HNCMs-N, **(C)** HNCMs-D//HNCMs-D, and **(D)** HNCMs-P//HNCMs-P.

The cycling stabilities of HNCMs-X//HNCMs-X supercapacitors were studied by a continuous GCD test at the current density of 1 A g^−1^. As shown in Figure 8, the capacitance retentions of HNCMs-X//HNCMs-X supercapacitors were all > 95.8% after consecutive 10,000 cycles, illustrating good energy storage stability. Additionally, these devices also displayed a steady coulombic efficiency > 96.1% after 10,000 cycles, demonstrating its high reversibility. Among the four HNCMs-X//HNCMs-X supercapacitors, the HNCMs-S//HNCMs-S symmetric supercapacitor displayed the most outstanding cycling stability (>98.4%) and coulombic efficiency (>99.0%) after 10,000 cycles.

## Conclusions

In conclusion, HNCMs with well-defined morphology have been synthesized by dual-template strategy employing CPC/PAA organic mesomorphous complexes as dynamic soft template, the *in situ*–generated silica as hard template, TEOS as silica source, and sucrose as carbon precursor. By tailoring the amount of PAA in the synthesis, four types of HNCMs with well-defined morphologies including submicrospheres (HNCMs-S), hexagonal nanoplates (HNCMs-N), dumbbell-like particles (HNCMs-D), and hexagonal microprisms (HNCMs-P) were successfully synthesized. Comparative study on the electrochemical properties of HNCMs with different morphologies showed that the HNCMs-S–based electrode exhibited the most outstanding specific capacitance of 233.8 F g^−1^ at the current density of 1 A g^−1^ due to the large surface area and well-defined hierarchically nanoporous structure. The symmetrical supercapacitor devices (HNCMs-X//HNCMs-X) were successfully assembled to testify the practical application of the HNCMs. Electrochemical test indicated that the HNCMs-S//HNCMs-S supercapacitor possessed a superior specific capacitance of 55.5 F g^−1^ at the current density of 0.5 A g^−1^ and higher energy density (7.7 Wh kg^−1^ at a power density of 250 W kg^−1^), the most outstanding rate capability and cycle ability. This research provides a strategy for controlled synthesis of HNCMs with well-defined morphology and gives theoretical guidance for preparing high performances of HNCMs and their related energy storage devices.

## Data Availability Statement

The original contributions presented in the study are included in the article/Supplementary Materials, further inquiries can be directed to the corresponding author/s.

## Author Contributions

LXi was responsible for the most of experiments and paper writing. KY associated with the assemble of the symmetrical capacitiors. JX provided advice for sloving the setback of the experiments. YZ guided some experimental running. LXu offered quite a few experimntal approaches. NL contributed some experimental ideas and modifies the whole paper. JD charged the whole framework of the paper. All authors contributed to the article and approved the submitted version.

## Conflict of Interest

The authors declare that the research was conducted in the absence of any commercial or financial relationships that could be construed as a potential conflict of interest.
