# Supplementary material for: Comparative Study on Supercapacitive Performances of Hierarchically Nanoporous Carbon Materials With Morphologies From Submicrosphere to Hexagonal Microprism
Source: Front Chem. 2020 Nov 17;8:599981. doi: 10.3389/fchem.2020.599981 (PMC7705105; doi:10.3389/fchem.2020.599981)
Supplement: Supplementary file 1 [file Data_Sheet_1.PDF]

Supplementary Material

MATERIALS AND METHODS

Chemicals and Materials

The chemical regents of hexadecylpyridinium chloride (CPC), hydrofluoric acid (HF, ≥40%), Poly(acrylic acid) (PAA) (average molecular weight = 240,000 g/mol, 25% solution in water), Tetraethylsiloxane (TEOS), sucrose and Ammonia used in this experiment were purchased from Alfa Aesar. Polytetrafluoroethylene (catalog number D-210C) was got from Daikin Fluorochemicals (China) Co., Ltd., Japan. Nickle foam was received from Liyuan New Materials Co., Ltd., Changde, China. All the chemical reagents were in analytical grade and used without further purification.

Synthesis of HNCMs

In a typical synthesis, 0.54 g CPC and a certain amount of PAA (4.0 g, 4.9 g, 5.3 g and 5.8 g) were dissolved in 30 mL deionized water under rapid stirring to obtain a transparent solution. Subsequently, 2.0 g ammonia solution (25 wt%) was added to the above solution with vigorous stirring. After 20 min of continuously stirring, 2.08 g TEOS and 4.28 g of sucrose were added to the emulsion under vigorous stirring. Next, the mixture was stirred at room temperature for 30 min, transferred into an autoclave and placed in an oven at 80 °C for 48 h. After the completion of the reaction, a brown milky suspension was obtained. The brown suspension was centrifuged, washed with deionized water, and dried at 70 °C for 12 h to obtain silica/sucrose composite as a brown powder. The as-synthesized brown composite was polymerized at 150 °C for 6 h, and calcined at 900 °C for 3 h under an argon flow to prepare carbon/SiO_2 composite. The heating rate was 2 °C min^{-1} below 600 °C and 5 °C min^{-1} above 600 °C. Finally, the as-synthesized silica/carbon composite was immersed in an excess amount of 10% aqueous HF solution (the molar ratio of SiO_2/HF=1:8) to remove silica, followed by washing with deionized water and vacuum drying at 50 °C to obtain hierarchically nanoporous carbon materials as black powder.

Materials Characterization

Field emission scanning electron microscopy (FE-SEM) images were obtained with Thermal scientific Apreo S LoVac instrument. Transmission electron microscopy (TEM) images measurement was conducted by using a JEOL JEM-2010 STEM/EDS microscope at an acceleration voltage of 200 kV, whereby a small drop of the sample was deposited onto a carbon-coating copper grid and dried at room temperature under atmospheric pressure. The powder X-ray diffraction (XRD) measurement was performed on a Rigaku Model D/max-2500 diffractometer, with Cu Kα radiation in the 2θ range of 10-80° with a step size of 0.02°. N_2 adsorption measurements were performed on a Micromeritics TriStar II 3020 sorption analyzer. Before measurements, the samples were dried under dry nitrogen flow at 120 °C for 12 h. The analytical data were processed by the Brunauer-Emmett-Teller (BET) equation for surface areas and by the Density Functional Theory (DFT) method by using nitrogen adsorption data for pore size distribution.

Preparation of HNCMs Based Electrodes and Electrochemical Property Characterization

To prepare the HNCMs based electrodes electrode, 80 wt% the HNCMs, 10 wt% acetylene black and 10 wt% polytetrafluoroethylene (PTFE) were well mixed to get slurry by adding ethanol as a solvent. The slurry was filled into the nickel foam substrate using a spatula, dried at 80 °C for 12 h, and then pressed at 16 MPa for 3 min in order to assure a good electronic contact between the nickel foam substrate and the active material. Finally, the as-prepared electrode was dried at 60 °C in vacuum for 24 h. The mass loading of working electrode was about 2 mg cm^{-2}. By varying the kind

of HNCMs, four HNCMs based electrodes denoted as HNCMs-S electrode, HNCMs-N electrode, HNCMs-D electrode and HNCMs-P electrode, respectively, were obtained.

The electrochemical property of the HNCMs based electrodes were test in a three-electrode system employing an Hg/HgO electrode was used as the reference electrode, a Pt foil were used as the counter electrode, and 6 M KOH as electrolyte. The electrochemical tests including cyclic voltammetry (CV), galvanostatic charge/discharge test (GCD) and electrochemical impedance spectroscopy (EIS) were performed on an electrochemical workstation (CHI 760E, chinstruments, Shanghai).

Assemble of HNCMs-X Based Symmetrical Supercapacitor and Supercapacitive Property Characterization

The symmetrical supercapacitor device was assembled by two HNCMs-X electrodes with the same size in a CR2025 type coin cell according to the order of electrode-separator-electrode (denoted as HNCMs-X//HNCMs-X supercapacitor). The electrolyte was 6 M KOH and the separator was a commercial polypropylene membrane. All the supercapacitor devices were assembled in air atmosphere. Then the CR2025 type coin cell was sealed with a pressure of 50 kg/cm^2 by using a MSK-110 sealing machine (Kejing Zhida Co., Ltd). The schematic illustration of HNCMs-X//HNCMs-X supercapacitors was shown in Scheme S1. The CV, GCD and EIS were also performed CHI 760E. The cycle life was measured by a supercapacitor test station (Neware BTS) on button cell supercapacitors assembled.

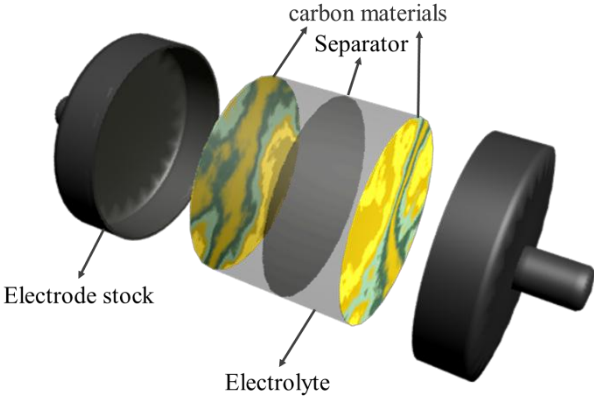

SCHEME S1 | The schematic illustration of HNCMs-X//HNCMs-X supercapacitors

Supplementary Figures

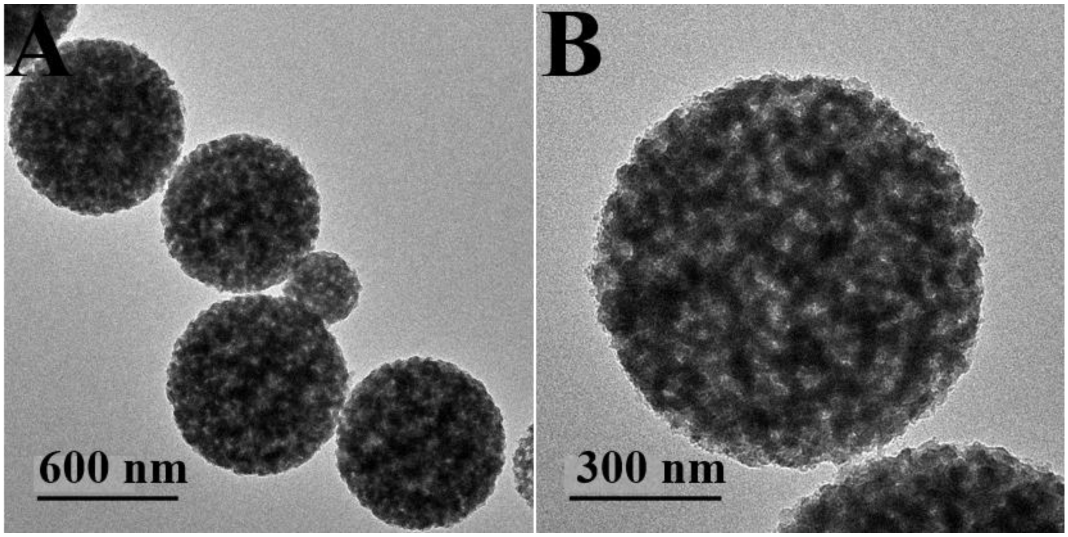

FIGURE S1 Transmission electron microscopy (TEM) images of the HNCMs-S sample synthesized with 4.0 g of PAA.

In order to characterize the interior texture of the HNCMs-S particle, the TEM test was carried out. It can be seen that the interior of the HNCMs-S particle exhibited obvious hierarchically nanoporous structure (Figure S1A,B). From the Figure S1B, it can be seen that the HNCMs-S composed of interstitial nanopores as well as mesopores, which were templated by cationic surfactant micelles CPC and polyelectrolyte PAA, respectively.

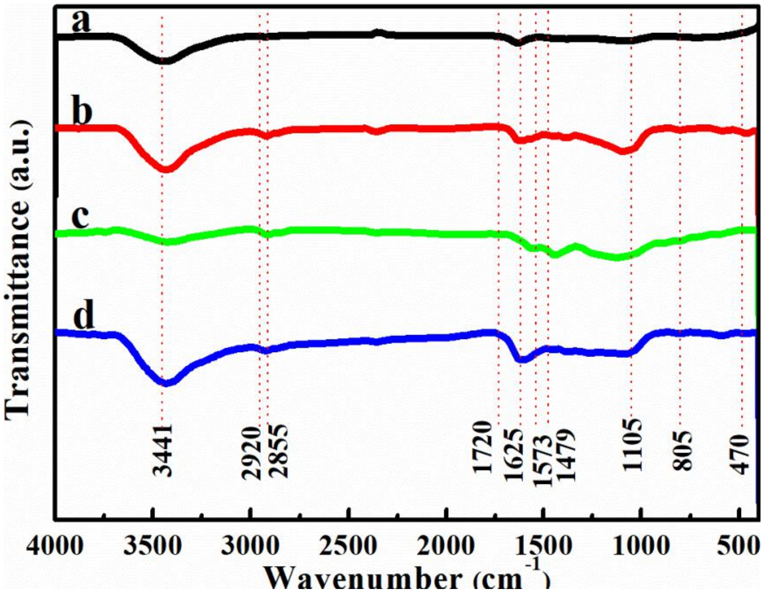

FIGURE S2 | FT-IR spectra of the HNCMs synthesized with different amount of PAA: HNCMs-S (pattern a), HNCMs-N (pattern b), HNCMs-D (pattern c) and HNCMs-P (pattern d).

In order to demonstrate the thorough removal of the silica species and organic templates, the FTIR spectra of the HNCMs sample with different PAA amount were performed. The spectra show that the HNCMs had similar chemical structures. As shown in Figure S2, the peak at 3441 cm^{-1} is ascribed to the stretching vibration peak of hydroxide radical, the characteristic peaks at 1105 cm^{-1} and 1625 cm^{-1} could be assigned to anti-symmetrical stretching vibrations of C-O and symmetrical stretching vibrations of C=C in sucrose oligomers, indicating the successful preparation of carbon framework. Moreover, the characteristic peak at 470 cm^{-1} and 805 cm^{-1} belonged to the silica species were almost disappeared. This indicates that the immersion of carbon/silica in HF solution could effectively remove the silica template. In addition, the stretching vibration peaks of methyl and methylene at 2855 cm^{-1} and 2920 cm^{-1}, the stretching vibration peak of carboxyl group at 1720 cm^{-1}, and the characteristic peaks at 1573 cm^{-1} and 1479 cm^{-1} attributed to the carboxylate asymmetric and symmetric –COO– stretching, derived from the templates of PAA and CPC organic mesomorphous complexe were almost unobserved. This further confirmed the complete removal of the organic PAA/CPC complex mesomorphous templates.

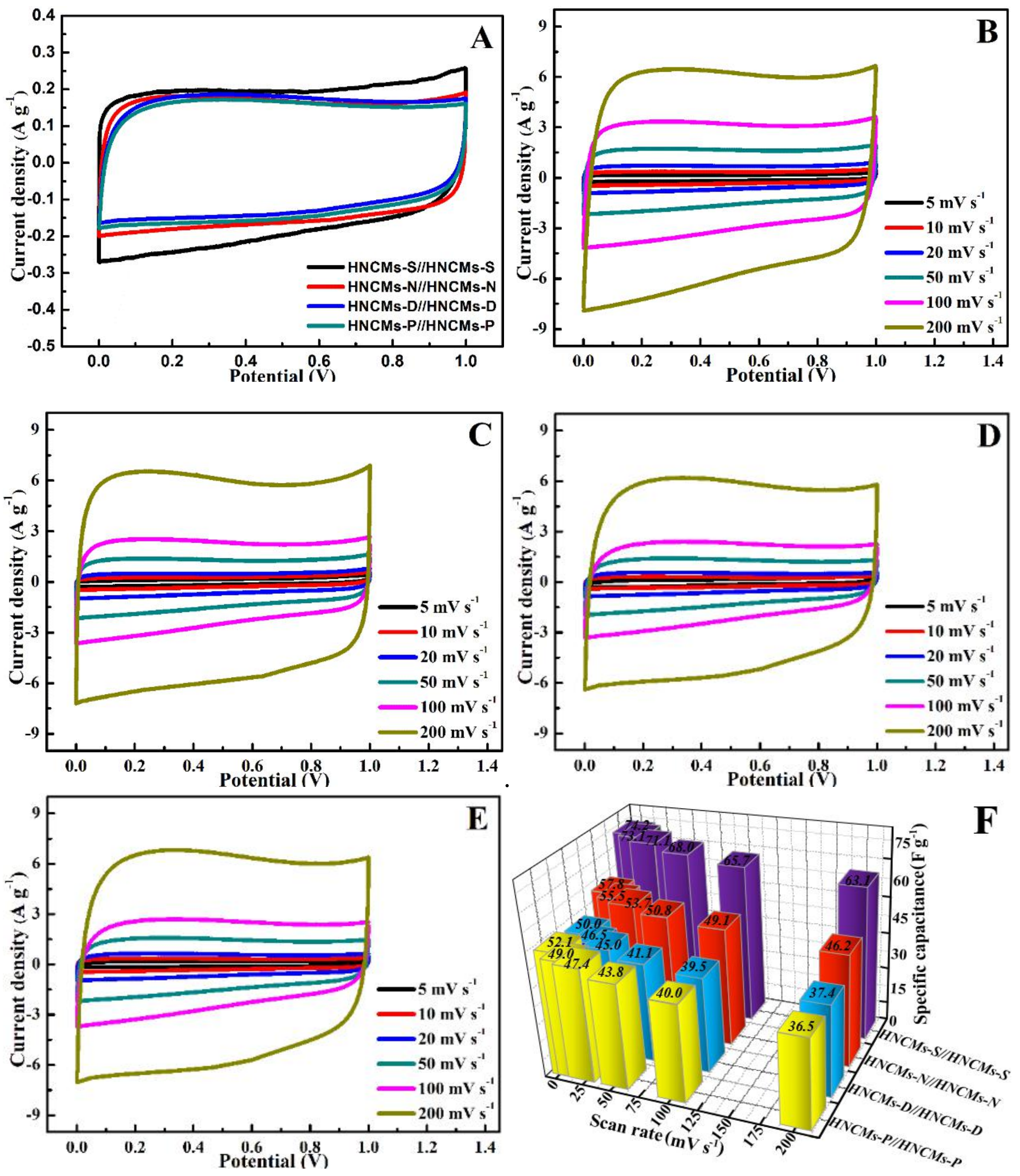

FIGURE S3 | (A) Cyclic voltammetry curves of HNCMs-X//HNCMs-X supercapacitors at scan rate of 5 mV s^{-1}, cyclic voltammetry curves of HNCMs-X//HNCMs-X supercapacitors at different scan rates: **(B)** HNCMs-S//HNCMs-S, **(C)** HNCMs-N//HNCMs-N, **(D)** HNCMs-D//HNCMs-D and **(E)** HNCMs-P//HNCMs-P, **(F)** Specific capacitance of HNCMs-X//HNCMs-X supercapacitors at different scan rates.

Figure S3A compares the cyclic voltammetry (CV) curves of the four symmetric supercapacitors performed at 5 mV s^{-1} with a potential range from 0 to 1 V. As displayed, the CV curves of the four HNCMs-based supercapacitors display similar, quasi-rectangular shape, indicating the ideal double layer capacitive behavior of these HNCMs. Furthermore, the response current of HNCMs-S//HNCMs-S supercapacitor was much larger than that of other three HNCMs-X//HNCMs-X supercapacitors due to the fast electrolyte ion transportation in the electrochemical interfaces. Figure S3B-S3E show the CV curves of all the HNCMs-X//HNCMs-X supercapacitors at the

scanning rate from 5 to 200 mV s^{-1}. It can be seen that the quasi-rectangular shape can be maintained even at a high scan rate of 100 mV/s. This indicates that the HNCMs-X//HNCMs-X supercapacitors have superior rate capability, which was attributed to that the hierarchical porous structure is beneficial to the ion diffusion. The specific capacitance of HNCMs-X//HNCMs-X supercapacitors based CV curves at different scanning rates can be calculated by the following formula (Zhao et al., 2014):

$$C_s=(I_a+I_c)/2M(\mathrm{d}V/\mathrm{d}t) \qquad \text{(S1)}$$

where C_s stands for specific capacitance of the supercapacitor (F g^{-1}), M is the total active material mass of the positive and negative electrode (g) and the dV/dt is the scan rate (mV s^{-1}). The results were summarized in Figure S3F. As shown, the specific capacitance for HNCMs-S//HNCMs-S supercapacitor is the highest among four samples, which reaches 74.2 F g^{-1} at the scan rate of 5 mV. The superior electrochemical performance can be attributed to the larger surface area and smaller particle size, which provide shorter diffusion path for electrolyte ions.

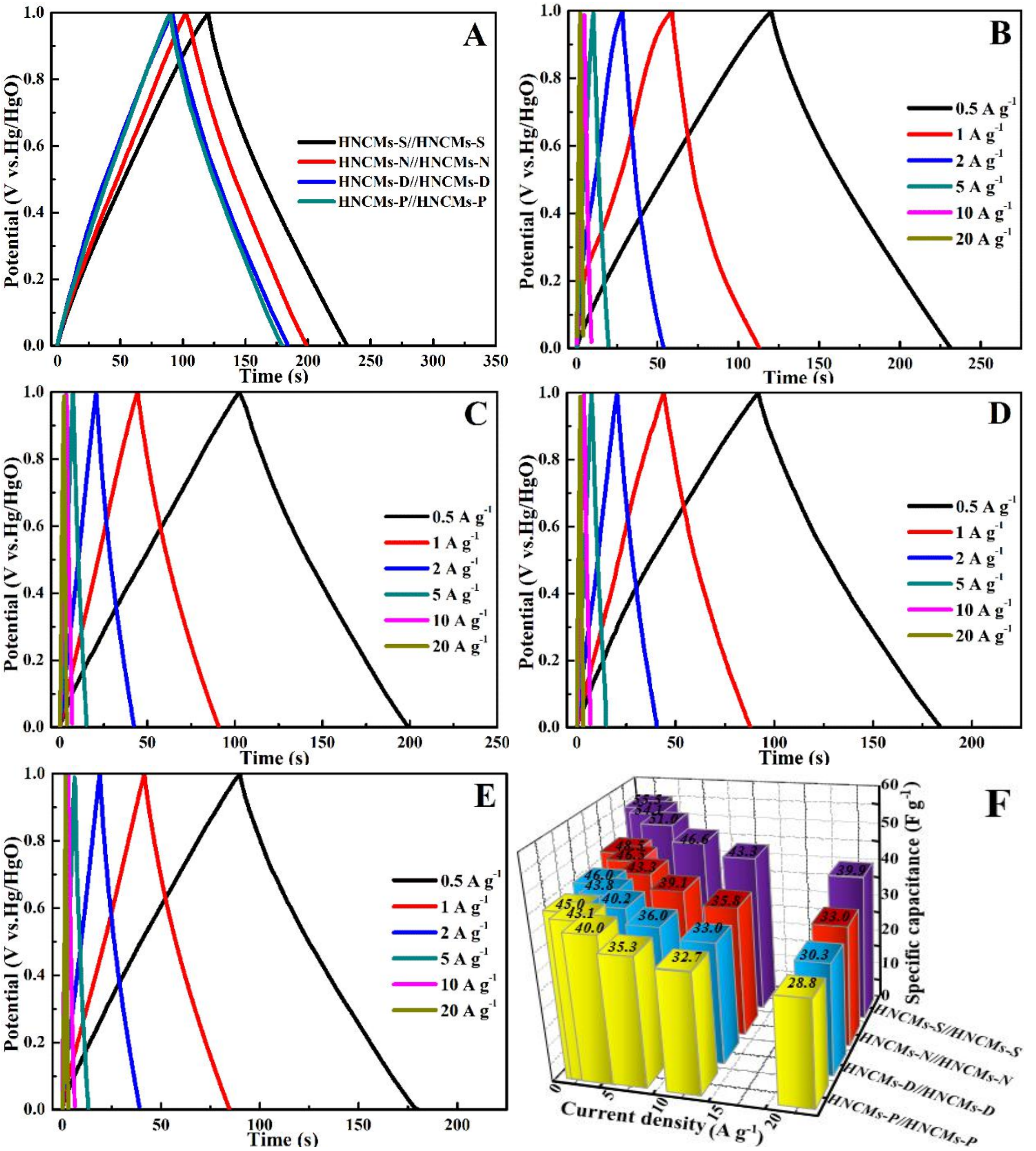

FIGURE S4 | (A) Galvanostatic charge and discharge curves of HNCMs-X//HNCMs-X supercapacitors at current density of 0.5 A/g, Galvanostatic charge and discharge curves of HNCMs-X//HNCMs-X supercapacitors at different current densities: **(B)** HNCMs-S//HNCMs-S, **(C)** HNCMs-N//HNCMs-N, **(D)** HNCMs-D//HNCMs-D and **(E)** HNCMs-P//HNCMs-P, **(F)** Specific capacitances of HNCMs-X//HNCMs-X supercapacitors at different current densities.

The galvanostatic charge/discharge (GCD) curves of the HNCMs-based supercapacitors at the current density of 0.5 A g^{-1} was compared. As shown in Figure S4A, all the GCD curves of the four HNCMs-X// HNCMs-X supercapacitors show approximately isosceles triangular shape, which implies an outstanding electrochemical reversibility. Obviously, the HNCMs-S// HNCMs-S supercapacitor has longer discharging time than other HNCMs-based supercapacitiors, revealing its high specific capacitance. The specific capacitance of HNCMs-X//HNCMs-X supercapacitors based GCD curve can be calculated according to the following equation (Sun et al., 2018):

$$C_m = I\Delta t/M\Delta V \qquad (2)$$

where C_m is specific capacitance (F g^{-1}), I stands for the constant discharging current (A), Δt is the discharging time (s) and M represent the total active material mass of the positive and negative electrode (g), ΔV stands for the potential range of the charge/discharge (V).The specific capacitance for HNCMs-S// HNCMs-S calculated based on the total mass of supercapacitor was 55.5 F g^{-1} at 0.5 A g^{-1}, which is much higher than HNCMs-N// HNCMs-N (48.5 F g^{-1}), HNCMs-D// HNCMs-D (46.0 F g^{-1}) and HNCMs-P// HNCMs-P (45.0 F g^{-1}) supercapacitors. The rate performance of HNCMs-based supercapacitior at current density range of 0.5-20 A g^{-1} (Figure S4B-S4E) exhibit the symmetrical triangular shapes, reconfirming the splendid coulombic efficiency. The specific capacitances calculated by GCD curves were summarized in Figure S4F. The specific capacitance of the HNCMs-S// HNCMs-S supercapacitor is higher than that of the other HNCMs-based supercapacitiors at all the current density, which is in consistent with CV results. In addition, the specific capacitance retentions are 71.9%, 68.0%, 65.9% and 64.0% for HNCMs-S//HNCMs-S, HNCMs-N//HNCMs-N, HNCMs-D//HNCMs-D and HNCMs-P//HNCMs-P supercapacitors, respectively. Obviously, the HNCMs-S//HNCMs-S supercapacitor showed the highest rate performance due to its superior hierarchical porous structure and high surface area.

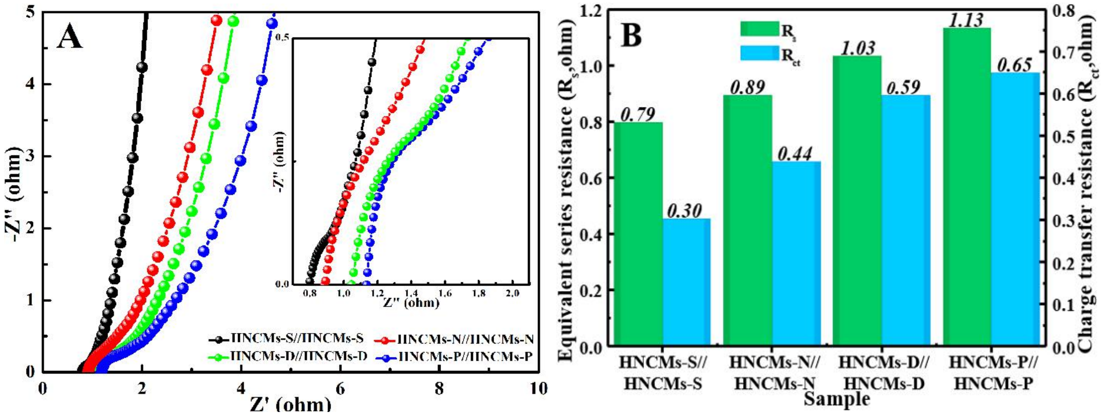

FIGURE S5 | (A) Nyquist plots of HNCMs-X//HNCMs-X supercapacitors and the inset image shows the magnified curve in the high frequency region. **(B)** R_s and R_{ct} values of HNCMs-X//HNCMs-X supercapacitors.

Figure S5A shows the Nyquist plots of HNCMs-X//HNCMs-X supercapacitors with a frequency range from 10^5 Hz to 10^{-2} Hz. As displayed, all the inset Nyquist plots of HNCMs-based supercapacitors show a similar shape, which composed of a depressed semicircle in high frequency region and a vertical line in low frequency regions. The results were displayed in columnar diagram in Figure S5B. Apparently, the HNCMs-S//HNCMs-S supercapacitor displays a better ion diffusion behavior. The relative lower internal resistance (R_s) of 0.79 Ω and smaller charge transfer resistance (R_{ct}) of 0.30 Ω than other HNCMs-X//HNCMs-X supercapacitors are attributed to the good conductivity of the HNCMs-S, high ion-diffusion efficiency of the hierarchical porous architecture.

References:

Sun, K. J., Zhang, Z. G., Peng, H., Zhao, G. H., Ma, G. F., and Lei, Z. Q. (2018). Hybrid symmetric supercapacitor assembled by renewable corn silks based porous carbon and redox-active electrolytes. *Mater. Chem. Phys.* 218, 229-238. doi: 10.1016/j.matchemphys.2018.03.022

Zhao, Q. L., Wang, X. Y., Wu, C., Liu, J., Wang, H., Gao, J., et al. (2014). Supercapacitive performance of hierarchical porous carbon microspheres prepared by simple one-pot method. *J. Power Sources* 254, 10-17. doi: 10.1016/j.jpowsour.2013.12.091
